# A multi-level system quality improvement intervention to reduce racial disparities in hypertension care and control: study protocol

**DOI:** 10.1186/1748-5908-8-60

**Published:** 2013-06-04

**Authors:** Lisa A Cooper, Jill A Marsteller, Gary J Noronha, Sarah J Flynn, Kathryn A Carson, Romsai T Boonyasai, Cheryl A Anderson, Hanan J Aboumatar, Debra L Roter, Katherine B Dietz, Edgar R Miller, Gregory P Prokopowicz, Arlene T Dalcin, Jeanne B Charleston, Michelle Simmons, Mary Margaret Huizinga

**Affiliations:** 1 Department of Medicine, Johns Hopkins University School of Medicine, 2024 East Monument Street, Suite 2-515, Baltimore, Maryland 21287, USA; 2 Department of Health Policy and Management|, Johns Hopkins Bloomberg School of Public Health, Baltimore, Maryland, USA; 3 Department of Medicine, University of Rochester Medical Center, Rochester, New York, USA; 4 Department of Epidemiology, Johns Hopkins Bloomberg School of Public Health, Baltimore, Maryland, USA; 5 Department of Family and Preventative Medicine, University of California San Diego School of Medicine, La Jolla, California, USA; 6 Department of Health, Behavior and Society, Johns Hopkins Bloomberg School of Public Health, Baltimore, Maryland, USA; 7 Community and Provider Advisory Board, Johns Hopkins Center to Eliminate Cardiovascular Health Disparities, Baltimore, Maryland, USA; 8 Department of Medicine, Vanderbilt University Medical Center, Nashville, Tennessee, USA

**Keywords:** Quality improvement, Hypertension, Health disparities, Pragmatic trial, Organizational culture, Community-based participatory research, Study design

## Abstract

**Background:**

Racial disparities in blood pressure control have been well documented in the United States. Research suggests that many factors contribute to this disparity, including barriers to care at patient, clinician, healthcare system, and community levels. To date, few interventions aimed at reducing hypertension disparities have addressed factors at all of these levels. This paper describes the design of Project ReD CHiP (Reducing Disparities and Controlling Hypertension in Primary Care), a multi-level system quality improvement project. By intervening on multiple levels, this project aims to reduce disparities in blood pressure control and improve guideline concordant hypertension care.

**Methods:**

Using a pragmatic trial design, we are implementing three complementary multi-level interventions designed to improve blood pressure measurement, provide patient care management services and offer expanded provider education resources in six primary care clinics in Baltimore, Maryland. We are staggering the introduction of the interventions and will use Statistical Process Control (SPC) charting to determine if there are changes in outcomes at each clinic after implementation of each intervention. The main hypothesis is that each intervention will have an additive effect on improvements in guideline concordant care and reductions in hypertension disparities, but the combination of all three interventions will result in the greatest impact, followed by blood pressure measurement with care management support, blood pressure measurement with provider education, and blood pressure measurement only. This study also examines how organizational functioning and cultural competence affect the success of the interventions.

**Discussion:**

As a quality improvement project, Project ReD CHiP employs a novel study design that specifically targets multi-level factors known to contribute to hypertension disparities. To facilitate its implementation and improve its sustainability, we have incorporated stakeholder input and tailored components of the interventions to meet the specific needs of the involved clinics and communities. Results from this study will provide knowledge about how integrated multi-level interventions can improve hypertension care and reduce disparities.

**Trial Registration:**

ClinicalTrials.gov NCT01566864

## Introduction

Critical health disparities exist between African Americans and their white counterparts in the United States. Cardiovascular disease accounts for more than one-third of the differences in life expectancy between African Americans and whites [1]. This disparity is largely attributed to hypertension and poor blood pressure control [1,2]. In population-based surveys, African Americans compared to non-Hispanic whites are more likely to have hypertension, are treated less often, and have lower rates of blood pressure control when treated [3,4].

The pursuit of remedies for these disparities between African Americans and whites has uncovered multi-factorial sources of the problem, including barriers to access, adherence and guideline compliance at the patient, provider, healthcare system, and community levels. The fundamental questions of which factors to address and how to address them are difficult to answer. Although interventions to date may address one or another source of disparities [5-7], few if any have intervened on multiple actors in the system: patients, clinicians, healthcare organizations and communities. Further, even successful interventions to reduce disparities may not be sustained past the end of the research funding. Interventions may ultimately fail when: the intervention was not adapted well to the organization where it was implemented; clinicians did not endorse the intervention; patients and their families were not actively engaged; or the communities where patients live posed so many challenges that patients could not maintain adherence to the intervention over time. Interventions are more likely to be sustained if they can answer such questions as: Do the patients, families, and clinicians understand the intervention? Is it relevant to what they care about? Does it address the adherence barriers they face? Does the intervention fit the local organization where it is being implemented?

The impracticality of standardizing every aspect of healthcare delivery makes the classic randomized controlled trial challenging to conduct in real world settings. Thus, we applied a pragmatic trial design to test whether racial disparities in blood pressure can be improved in six community practices in Baltimore, Maryland. Using this pragmatic approach with concepts from Community-Based Participatory Research [8], we seek to show effectiveness in uncontrolled, routine clinical care [9]. This project realizes the importance of adapting the intervention to specific characteristics of the clinic sites, taking into account clinician attitudes about the intervention, directly addressing patient adherence barriers, and engaging the community to make the intervention more sustainable when the research study is over.

## Methods

### Study design and specific aims

#### Specific aims

This ongoing study has three specific aims, which are reflected in Table 1 with associated hypotheses and primary outcome measures. First, we are implementing a multi-level system quality improvement intervention to reduce racial disparities in blood pressure control via three multi-level interventions designed to standardize the measurement of blood pressure, provide patient care management services, and offer expanded provider education resources.

**Table 1:** Project ReD CHiP’s specific aims, hypotheses and main outcome measures

**Specific aims**	**Hypotheses**	**Process and outcome measures**
To perform a multi-method, staged quality improvement intervention (better blood pressure measurement, patient care management and provider education) to increase guideline concordant hypertension care and to reduce racial disparities in blood pressure control.	• Better blood pressure measurement and better blood pressure data will lead to less clinical inertia and, ultimately, better blood pressure control and less racial disparities.	• % of patients with controlled BP and % of patients with uncontrolled BP with medication titration in the last 3 months
	• Each intervention will have an additive effect and the use of all three interventions will result in a higher percentage of patients receiving guideline-concordant hypertension care and a greater reduction in racial disparities than any single combination or any combination of two interventions.	• % of patients with controlled BP and racial disparity in controlled BP
	• The care management intervention will have greater net improvement in blood pressure control at the clinic level than the provider education intervention. The provider education will have an additive and greater effect when implemented after the care management intervention than when employed without the care management intervention.	• % of patients with controlled BP and racial disparity in controlled BP
To determine the association of organizational functioning and organizational cultural competence with guideline concordant hypertension care and racial disparities in blood pressure control.	• Clinics will reflect their local surroundings and clinics with a higher percentage of minority persons will have lower organizational functioning.	• % of patients with BP control, stratified by race, compared across clinics
	• Clinics with greater organizational cultural competence will have greater guideline concordant hypertension care and less racial disparities in blood pressure control.	• Degree of racial disparity
To determine the association between organizational functioning and organizational cultural competence at the clinic and system level with the implementation and success of the quality improvement interventions.	• Clinics with higher organizational functioning will have a higher rate of implementation and more blood pressure control and reduction in racial disparities than clinics with lower organizational functioning.	• Degree of implementation for each of the interventions
	• Clinics with greater organizational cultural competence will have a higher rate of implementation and more blood pressure control and reduction in racial disparities than clinics with less cultural competence.	• Degree of implementation for each of the interventions

Abbreviations: *%* percent; *BP* blood pressure.

Second, we aim to determine the association of organizational functioning and cultural competence at the clinic level (as perceived by providers and staff) with improvements in guideline concordant hypertension care and reductions in racial disparities in hypertension. Third, we seek to examine the relationship of organizational functioning and cultural competence with implementation success and effectiveness of each of the interventions. During the course of this project we will also examine the relationship of the interventions with improvements in patient level factors such as knowledge, attitudes, experience, activation levels, and medication adherence.

While we expect that each intervention will increase guideline-concordant hypertension care, we hypothesize that the combination of all three interventions will result in the greatest improvement in hypertension care and the largest reduction in racial disparities in blood pressure control. We further hypothesize that organizational functioning and cultural competence will be associated with guideline-concordant care and smaller racial disparities at baseline, and that higher scores on these organizational characteristics will be associated with more effective interventions, improvements in patient outcomes, and reductions in disparities over time.

**Figure 1:**
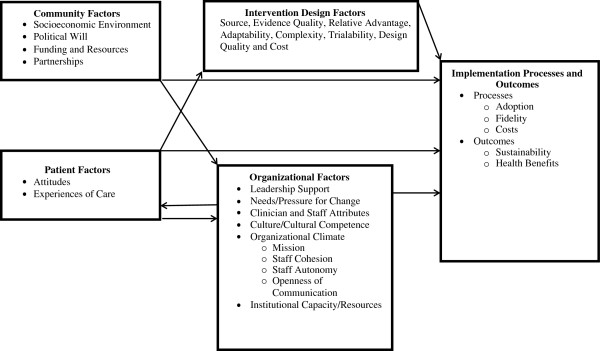
Project ReD CHiP’s conceptual model.

Project ReD CHiP (Reducing Disparities and Controlling Hypertension in Primary Care) is one of three research projects in the Johns Hopkins Center to Eliminate Cardiovascular Health Disparities, which is one of 10 NIH-funded Centers for Population Health and Health Disparities (CPHHD) [10]. The conceptual model we used to design Project ReD CHiP recognizes that its implementation and outcomes are influenced not only by the three interventions but also by patient, provider, organizational and community factors (Figure 1). This model is adapted from the works of Shediac-Ritzkallah and Bone [11], Simpson [12], and Damschroder *et al.*[13]. In addition to using a multi-method approach to address these factors, a Community and Provider Advisory Board formed of local stakeholders including political leaders, healthcare providers, patients, faith community representatives, and individuals from various community organizations has provided guidance throughout the project. Project ReD CHiP was approved by the Johns Hopkins Institutional Review Board, protocol number 00037622.

#### Study populations and settings

We are implementing Project ReD CHiP from 2010 to 2015 in six Johns Hopkins Community Physician (JHCP) primary care practices in the Baltimore, Maryland metropolitan region (Table 2). JHCP is a network of over 35 primary care and specialty practices serving the state of Maryland and greater Washington, D.C. region. JHCP has a tradition of innovation in health services delivery and has more than 1,000 employees and 370 physicians providing care for more than 230,000 patients annually. The clinic sites involved in Project ReD CHiP are located in both Baltimore City and Baltimore County and four of the practices are located in medically underserved areas. The six sites were chosen because they are community-based and serve patients from a variety of socio-demographic backgrounds. These practices were recruited with support from JHCP organizational leadership.

**Table 2:** Description of study clinics

	**Site A**	**Site B**	**Site C**	**Site D**	**Site E**	**Site F**
**Clinic Characteristics**						
Primary care providers, n	9	11	11	5	3	6
Patients, n	7,755	4,733	14,887	3,681	5,628	6,161
AA patients, %	65.6	90.1	23.3	18.4	17.7	20.2
White patients, %	28.0	4.4	68.9	77.7	73.3	72.6
AA patients with HTN, n	2,940	2,777	1,493	359	331	593
AA patients with uncontrolled HTN, %	33.1	38.8	29.7	40.1	40.5	42.8
White patients with HTN, n	705	80	4,209	1,362	650	1,477
White patients with uncontrolled HTN, %	29.2	33.9	24.3	34.9	37.1	30.6
**Local Characteristics**						
Medically underserved area^+^	Yes	Yes	Yes	No	Yes	No
Median income (in 2011 $US)^*^	$47,472	$36,652	$58,488	$50,459	$47,472	$99,155
% Below poverty line^*^	19.0	21.0	8.9	10.6	18.2	7.8
% Employed^*^	55.5	54.8	70.7	60.3	59.0	64.4
% Population AA^*^	71.7	59.1	19.5	16.9	34.1	27.6
% High school grad or equivalent^*^	81.4	76.9	85.6	78.7	78.2	92.7
% Vacant housing units^*^	16.7	19.6	6.7	10.6	14.5	5.6

+ By site address.

* By Zip Code Tabulation Area, American Community Survey, 2007–2011. The zip codes representing the residences of the majority of the patients at each site are included.

Abbreviations: *AA* African American, *n* number, *US* United States.

The clinic sites involved in Project ReD CHiP include 45 internal medicine, family practice and medicine/pediatric primary care providers (PCPs) that care for approximately 42,845 patients. Medical assistants (MAs; n = 50), registered dietitians (RDs; n = 3), and doctors of pharmacy (PharmDs; n = 4) also have important roles in this project. While individual PCPs can choose not to participate in the interventions, patient data from these providers will be included in the overall clinic measures as this is a multi-level system quality improvement intervention.

#### Interventions

Project ReD CHiP includes three primary interventions: improvement of blood pressure measurement using an automated device and a standardized protocol; care management services provided by embedded PharmDs and RDs; and provider education, communication training, and individualized provider audit and feedback (Table 3). We responded to stakeholder input and also specifically designed each of the interventions in this multi-method project to target factors known to contribute to hypertension disparities. Components of each intervention were additionally tailored to address disparities at patient, provider, and clinic levels (Table 4). Each intervention is discussed in detail below.

**Table 3:** Features of Project ReD CHiP’s interventions

	Intervention*	**Blood pressure measurement**	**Care management**	**Provider education**
	**Goal**	• Improve accuracy and reliability of blood pressure measurement and reduce clinical uncertainty	• Add RDs and PharmDs to primary care teams to deliver culturally-sensitive patient education, promote self-management behaviors and improve access and team functioning	• Incorporate best practices in physician education by assessing PCP needs, delivering an interactive program to provide practical communications skills training and providing performance data feedback on blood pressure control among patients stratified by race/ethnicity
	**Participants and clinics**	• Medical assistants and providers	• Eligible patients (SBP ≥ 140 and/or DBP ≥ 90 mmHg) attending the clinic; providers and clinic staff in referral process	• Providers
		• All six participating JHCP clinics	• All six participating JHCP clinics; staggered roll out between 2012-2015	• All six participating JHCP clinics; staggered roll out between 2012-2015
	**Rationale**	Errors resulting from suboptimal blood pressure measurements can influence treatment decisions [14-16]	• Two systematic reviews of quality improvement strategies for hypertension management show team change interventions including assignment of some responsibilities to health professional other than provider result in largest blood pressure reductions [17,18]	• Participatory decision making style is associated with higher patient satisfaction, continuity of care, improved self-care behaviors and greater adherence to medications [11,19-22]
		• Standardizing and improving reliability of blood pressure measurements may improve PCP confidence in measures and reduce clinical inertia for treatment		• Audit and feedback approaches have been associated with improved quality metrics
		• Provides standardized measurement for other two interventions		
**Level of intervention**	**Patient**	• Providing posters in check-in and exam areas that demonstrate appropriate positioning and give reasons for new process	• Participating in three care management sessions, totaling two hours	• Promoting patient engagement indirectly by enhancing providers’ patient-centered communication and participatory decision-making skills
			• Intervening on lifestyle: exercise, weight loss, DASH diet, medication adherence	
	**Provider and staff**	• Educating providers and medical assistants about proper blood pressure measurement through didactic and skills practice	• Referring eligible patients to care management team	• Providing audit and feedback via race-stratified hypertension dashboard and web based video training targeting communication skills that promote patient adherence
			• Receiving reimbursements for panel review of eligible hypertension patients	
	**Clinic**	• Introducing tools to facilitate adherence to recommended techniques (e.g., Omron HEM-907XL)	• Embedding RDs and PharmDs in clinics as part of the provider support team	• Building hypertension dashboard on existing JHCP provider dashboard
		• Redesigning patient intake protocols (proper patient positioning and multitasking during Omron use)		• Contracting with JHCP IT team to develop and refine hypertension dashboard
**Stakeholder input**	**Patients/ community**	• Suggested posters in exam rooms to explain new process	• Recommended specific educational materials and suggested changes to language, layout and images	• Provided suggestions to make patient stories more realistic for communication skills program
	**Medical assistants**	• Focus groups informed intervention development and implementation plan	• Focus groups informed intervention development and implementation plan	N/A
		• Identified and trained Master Trainers and Super-Users at each clinic to support adoption of devices		
		• Disseminated time-saving techniques developed by medical assistants to all sites		
	**Provider**	• Focus groups informed intervention development and implementation plan	• Focus groups informed intervention development and implementation plan	• Focus groups informed intervention development and implementation plan
		• Directed interviews to assess organizational culture	• Directed interviews to assess organizational culture	• Directed interviews to assess organizational culture
		• Identified JHCP provider champion	• Identified JHCP provider champion	• Identified JHCP provider champion
	**JHCP leadership**	• Directed interviews to assess organizational culture	• Directed interviews to assess organizational culture	• Directed interviews to assess organizational culture
	**Johns Hopkins Health Care (JHHC)**	N/A	• Modified existing care manager job description for RDs • Subcontract with JHHC to hire and fund study RDs	N/A

* All of the interventions aim to improve blood pressure control and reduce disparities in blood pressure; all introduce organizational change.

**Table 4:** Multi-level disparities tailoring in Project ReD CHiP’s interventions

		**Blood pressure measurement**	**Care management**	**Provider education**
	**Disparities Specific Rationale**	• Clinical uncertainty is believed to be a major contributor to healthcare disparities [23]	• Racial disparities in blood pressure control are due, in part, to poorer adherence to medications [24], limited access to healthful foods [25] and poor quality diets [26]	• PCPs use less patient-centered communication in visits with African American patients [27,28]; patients with low health literacy ask their physicians fewer questions about medical care issues [29]
		• Standardization of healthcare processes may reduce clinical uncertainty and variations in care [30]	• 2Motivational interviewing is effective at promoting health behavior change [31,32], medication adherence [33] and health outcomes [31-33] in African Americans	• The combination of cultural competency training and race-stratified performance reports increases clinician awareness of racial disparities in care [34]
			• Delivery of culturally and linguistically-tailored health information increases acceptability of interventions in minority populations [35]	• PCP communication skills training improves patient-reported outcomes and blood pressure control in ethnic minorities and poor persons [36]
**Disparities Tailoring**	**Patient**	• Poster messages and images reduce patient anxiety and promote trust	• Educational materials culturally and linguistically tailored	• Patient scenarios include individual and environmental determinants of disparities and demonstrate methods to address them
			• Motivational interviewing enhances patient engagement and addresses individual determinants of disparities	
			• Community resource guide addresses environmental determinants of disparities	
	**Provider and Staff**	• Re-training videos use local staff as role models	• Care managers enhance providers’ ability to address patients’ complex psychosocial needs by providing additional counseling and support	• Dashboard increases provider awareness of disparities
				• Communication skills training enhances provider participatory skills leading to increased patient trust and engagement
	**Clinic**	• Patient posters provide culturally and linguistically tailored communication	• Availability of phone contacts and flexible appointment times enhances access	• Financial incentives reward providers for reviewing disparities data
			• Financial incentives encourage providers to make referrals	

During the project’s development phase, physicians, nurse practitioners and MAs participated in baseline focus groups to discuss the feasibility and acceptability of the planned interventions. Research staff also attended clinic meetings and provider retreats and hosted webinars and workshops to hear feedback from individuals working in the clinics. At each site, organizational leaders as well as senior and practice level administrators completed semi-structured interviews with research staff. The results from these activities helped to shape the interventions. We will conduct follow-up focus groups and semi-structured interviews at the end of implementation to help increase the long-term effectiveness and sustainability of the interventions.

#### Blood pressure measurement

The blood pressure measurement intervention aims to improve blood pressure control and reduce disparities by improving the accuracy and reliability of clinical measurements. Previously published studies have shown that adherence to recommended blood pressure techniques in clinical practice are suboptimal and that errors in measurements can influence clinicians’ treatment decisions [14-16]. Additionally, many clinical blood pressure measurements are associated with terminal digit bias, the phenomenon where measurements are rounded off, commonly to zeros [37,38].

In the blood pressure measurement intervention we provided each PCP/MA team at the clinics with an automated blood pressure measurement device (Omron HEM-907XL). This device features programmable settings that only inflate the blood pressure cuff after a timed, three minute rest period (adjusted from the standard five minute countdown to accommodate concerns regarding workflow) and then automatically obtains three consecutive measurements, each separated by 30 seconds. The device displays the mean of the three measurements and the MA records the mean value in the electronic medical record (EMR). In addition to facilitating the use of pre-measurement rest periods and obtaining sequential measurements, the use of these devices eliminates the terminal digit bias often associated with manual measurements and provides PCPs with valid and reliable blood pressure readings for every patient. As clinical uncertainty is believed to be a significant contributor to healthcare disparities [23], the introduction of automated blood pressure devices ensures the standardization of an important healthcare process, which may reduce clinical uncertainty and variations in care [30].

At initial rollout, hypertension specialists held education sessions at each site to introduce the devices and provide clinicians and staff with evidence for the importance of accurate blood pressure measurements. Research team members provided on-going site level support for 15 months as the staff became accustomed to the devices. We placed culturally and linguistically tailored posters explaining the new procedure for blood pressure measurement throughout the clinics to promote patient engagement. To improve sustainability of the intervention, the device maintenance and personnel training responsibilities are being transferred to the organization’s quality improvement department and key staff at individual clinic sites.

#### Care management

The care management intervention seeks to address blood pressure management by providing patient education, promoting self-management behaviors, and introducing organizational change. Poor adherence to medications [24], limited access to healthful foods [25], and poor quality diets [26] contribute to racial disparities in blood pressure control. The care management team aims to reduce disparities and achieve guideline concordant care through the promotion of self-management skills and medication titration.

**Figure 2:**
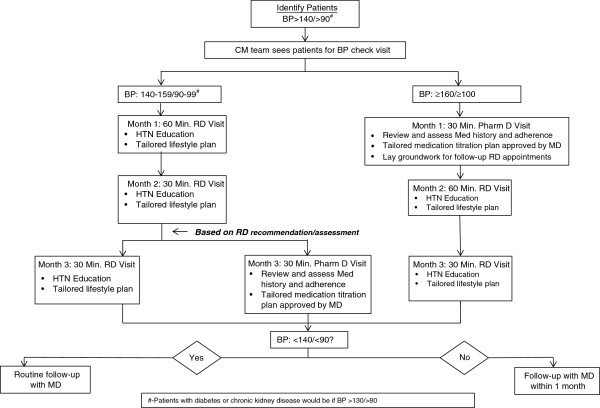
Project ReD CHiP’s flowchart for patients in care management.

Patients with a diagnosis of hypertension (ICD9 codes) and with their most recent blood pressure ≥140/≥90 mmHg are eligible for care management services. Care managers (either RDs or PharmDs) review the clinic’s electronic patient registry, which abstracts data from the practice’s EMR, to identify eligible patients to outreach via telephone. Providers in the clinic also refer eligible patients directly to the care managers for care management services. Eligible patients receive three sessions totaling 120 minutes with an RD and/or a PharmD (Figure 2). The RD covers lifestyle behaviors related to the management of hypertension including the DASH diet, weight loss, and exercise. The PharmD addresses issues related to medication adherence and titration, if necessary. Embedding care managers in the clinic allows for additional patient counseling and enhances providers’ ability to address patients’ complex needs.

Recognizing the effectiveness of motivational interviewing in promoting health behavior change [31,32], medication adherence [33], and health outcomes [31-33] in African Americans, our care managers utilize motivational interviewing techniques to assess patients’ knowledge of hypertension, current self-management practices, barriers to self-management, and individual preferences in managing hypertension. Additionally, the care managers provide education and recommendations specific to patients’ needs, preferences, and cultural context through the use of customizable, literacy-sensitive modules about blood pressure management. The care managers also utilize a community resource guide to help address the environmental determinants of disparities. Delivery of culturally and linguistically tailored health information increases the acceptability of interventions in minority populations [35].

#### Provider education

The provider education intervention aims to improve blood pressure control and reduce disparities by delivering individualized performance feedback and introducing additional provider-tailored educational resources. It has been shown that PCPs use less patient-centered communication in visits with African American patients [27,28], and patients with low health literacy ask their physicians fewer questions [29]. In developing this intervention, we recognized that the combination of race stratified performance reports and cultural competency training increases clinician awareness of disparities in care [34] and thus incorporated these strategies.

Through this intervention providers are given access to a web-based dashboard that imports clinic measurements and patient information from the EMR. The dashboard increases providers’ awareness of disparities by identifying the percentage of their patients achieving guideline-concordant hypertension control overall as well as among their African American and white patients.

To advance providers’ patient-centered communication skills we developed a training website containing a series of ultra-brief (approximately 30 second) video demonstrations of targeted skills using simulated patient-provider interactions. A pilot study using this approach found that clinicians reported significant changes in routine use of demonstrated skills even after less than 15 minutes of exposure to the website [39]. Moreover, in earlier work we found that providing clinicians with communication skills training can reduce disparities and improve patient-reported outcomes and blood pressure control in ethnic minorities [36]. The videos illustrate a variety of assessment and partnership skill examples to improve the patient-provider encounter and promote patient adherence. A variety of patient scenarios are presented and the videos demonstrate methods to help providers address individual and environmental determinants of disparities. Prior to viewing the video clips, providers complete a self-assessment regarding their use of specified communication behaviors during visits with uncontrolled hypertensive patients. Immediately after viewing the skills videos, they are asked to complete a short survey regarding their intention to use the demonstrated communication techniques. Six months after their first use of the website, physicians are contacted again and asked to report on their use of specified communication behaviors and encouraged to return to the website.

### Data collection, outcome measures, and statistical analysis plan

#### Data collection

Prior to implementation, PCPs and MAs at each clinic completed a ‘motivation survey’ to assess several areas of focus relevant to the acceptance and implementation of the interventions. The areas of focus included respondents’ readiness to change; perception of barriers to safety/quality and addressing healthcare disparities; perception of workflow and organizational stress; medication prescription practice; and how respondents felt the interventions would affect the care they provide to their patients. To better assess organizational functioning and examine how perceptions of the organization’s teamwork and safety culture could influence the interventions, we created an ‘organization survey’ by adding questions to the Safety Attitude Questionnaire [40], which JHCP administers annually to all providers and staff. These survey responses have been used to describe the baseline organizational environment at each of the six clinic sites.

In the care management intervention, after patients attend three visits with the care managers they will complete surveys to assess their experiences with the program. Additionally, at two time points throughout the study we are conducting a patient survey at two of the participating clinics. We will anonymously survey 210 hypertensive patients in the waiting room at each of the selected sites. The responses will help assess the impact of the intervention on patient-reported outcomes, including patient knowledge, attitudes, experiences of care, and self-reported behaviors at each site.

#### Outcome and process measures

We are retrospectively extracting a range of process and outcome measures (already collected by the clinics) from JHCP’s EMRs (Table 5). We are collecting aggregate data at the system, clinic and provider levels. Data will be expressed as an aggregate percentage for a time period of one week, with the denominator equal to the number of patients seen in clinic by all PCPs that week and the numerator equal to the number of patients that achieved the measure as defined in Table 5. Race/ethnicity, gender, age, and presence of other chronic illnesses (diabetes, chronic kidney disease, coronary artery disease) categories will be extracted and the aggregate data will be stratified by these categories. Race/ethnicity is self-reported at the initial clinic visit.

**Table 5:** Process and outcome measures used in Project ReD CHiP

**Type**	**Measure+**	**Notes and definitions**
Primary process measure	% with uncontrolled BP with medication titration in last 3 months	Uncontrolled BP ≥140/≥90 (or ≥130/≥80 if DM or chronic kidney disease); titration can be dose increase or medication change/addition
Secondary process measures	% with BP measure in EMR in last 12 months	
	% with history of pre-HTN with BP measure in last 12 months	Two clinic BPs ≥120-<140≥80-<90; not on HTN medication
	% with HTN with measure in last 6 months	Two clinic BP ≥140/≥90 OR use of a HTN medication OR ICD9 diagnosis (401.xx)
	% with HTN on a thiazide diuretic	
	% with HTN and DM on ACE-I or ARB	DM defined by ICD9 code 250.xx
	% with lipid panel performed in last 5 years^*^	May not be possible as EMR only in all clinics since March 2006
	% with BP≥135/≥85 screened for DM in last 5 years^*^	May not be possible as EMR only in all clinics since March 2006
Implementation uptake process measures	% with BP measured with OMRON	
	% of SBP and DBP measures ending in zero	
	% of eligible patients enrolled in care management	
	% completion rate for those enrolled in care management	
	Provider behavioral intention related to assessment and partnership skills	Measured at completion of the website review and at 6 months post-intervention
	Change in provider self-reported use of assessment and partnership behaviors	Measured before the website review and at 6 months post-intervention
	% of providers receiving web training at each site	Measured at 6 months post-intervention
	Provider self-reported use of HTN dashboard	Measured at 6 months post-intervention
Primary outcome measure	% with controlled BP	Controlled BP defined as <140/<90 (or <130/<80 if DM or chronic kidney disease present)
Secondary outcome measures	% with controlled LDL	Controlled LDL defined as <130 (or <100 if DM or coronary artery disease)
	% with controlled HDL	Controlled HDL defined as >50 in women or >40 in men
	% with controlled triglycerides	Controlled triglycerides defined as <150
	% with controlled A1C	Controlled A1C defined as <7.0
	Mean SBP, DBP, LDL, HDL, triglycerides, A1C	

+Data are aggregated in one week intervals at baseline, during and after intervention roll-out for a minimum of 24 weeks.

*These measures will only be performed in clinics that have had the EMR system in place for at least 5 years.

Abbreviations: *%* percent, *BP* blood pressure, *DM* diabetes mellitus, *EMR* electronic medical record; *HTN* hypertension, *ACE-I* angiotensin converting enzyme inhibitor, *ARB* angiotensin receptor blocker, *SBP* systolic blood pressure, *DBP* diastolic blood pressure, *LDL* low density lipoprotein, *HDL* high density lipoprotein, *A1C* glycated hemoglobin.

#### Statistical analysis plan

We will use Statistical Process Control (SPC) charting of measures over time to determine if there is a change at the system level after the implementation of the intervention and how long after implementation the change is evident [41,42]. SPC is a set of statistical methods based on the theory of variation that can be used to make sense of any process or outcome measured over time. Data will be visually inspected in a graphical display of a p-chart (for displaying proportion per time period). In this case, the x-axis will be each week of data collection (total of 24 weeks in the baseline data collection) and the y-axis will be the percent of patients seen that week with controlled blood pressure. We will distinguish normal variation from special causes (unusual changes in the pattern of data that can be assigned to a specific cause) using a range of statistically-driven tests, including: one value outside the control limits; two of three consecutive values above or below the mean and more than two standard deviations away from the mean; eight or more values falling above or below the mean; or six or more values in a row steadily increasing or decreasing (that is, showing a trend) [42].

We are also collaborating with health economists at our institution and other CPHHD centers to design and conduct a collection of cost-effectiveness analyses from health system, patient and societal perspectives, based on data from several of the ongoing interventions currently being studied by the CPHHD. We intend to capture program, patient and spillover healthcare costs, and cost offsets. The program perspective will determine the budgetary impact of implementing the interventions. Both patient and health system perspectives measure factors impacting individuals’ ability to adhere to the protocol. The success of interventions depends, in large part, on this. Additionally, interventions may have significant spillover effects, such as additional costs or cost offsets, on the healthcare system, which could impact the sustainability of the program within study sites and the likelihood of broader dissemination.

#### Trial status

**Figure 3:**
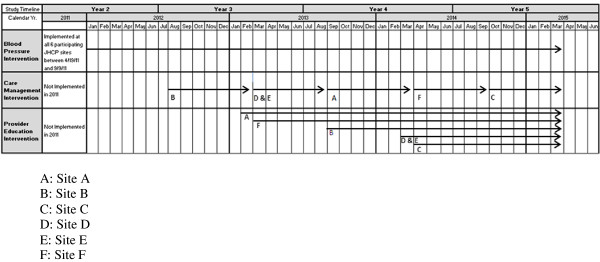
Project ReD CHiP’s anticipated intervention timeline.

The blood pressure intervention was introduced to all six clinic sites by September 2011 (Figure 3). In three of the clinics, the care management intervention starts six to nine months before the provider education intervention and in the other three clinics the care management intervention starts six to nine months after the provider education intervention. At the time of this writing, care managers have been embedded in one clinic site and are beginning to expand their services to two other sites. Providers at two of the sites have access to the dashboard and have seen the online communication training videos. We will continue to stagger the introduction of the care management and provider education interventions to the remaining clinics over the next two years.

## Discussion

This study aims to reduce racial disparities in blood pressure control and improve guideline concordant hypertension care by implementing a multi-level system quality improvement intervention. Because we have applied a pragmatic trial design, our interventions take place within existing clinic practices. In this discussion, we describe the changes we made to the intervention design to meet the needs of local settings and the lessons we have learned so far while implementing the projects.

We worked collaboratively with each clinic site as well as with leaders from the JHCP organization to improve the design of the interventions. Through focus groups and directed interviews, we learned about their concerns regarding certain components of the proposed interventions and we made changes to the intervention design prior to implementation based on their feedback. For example, to avoid interference with patient flow, clinic staff requested that there be at least one blood pressure device for each PCP. They felt that the care management services should have cultural and community relevance. We also learned that physicians preferred the dashboard to be developed in similar format and delivery to existing provider dashboards at JHCP. We responded to each of these suggestions and these changes were incorporated into the intervention design.

In addition to adapting certain elements of the intervention in direct response to specific clinic needs, it has also proven important to work with local champions throughout the implementation process. Prior to introducing the automated blood pressure devices, we identified one MA at each site as a ‘super-user’ These MAs not only encouraged their co-workers to follow the new protocol for measuring blood pressure, but they also served as liaisons between the research and clinic staff. In the care management intervention, we chose to have PharmDs who were already familiar with the clinical practices serve as part of the intervention team. They suggested that a triage system be created that would allow the patients with the most uncontrolled blood pressures to see the PharmD first to focus on issues of medication adherence. Their knowledge of the patient populations served at each clinic and their established relationships with clinic staff helped to improve uptake of the care management intervention.

We introduced the interventions in the context of other studies and other health system changes that were taking place at the clinics. Although we took steps in the development phase to incorporate site-level feedback, understand the organizational climate, and anticipate barriers to change, certain site level factors required us to make adjustments after initial implementation of the interventions. For example, in response to challenges with patient recruitment in the care management intervention at the first clinic, we adjusted our scheduling approach to better meet patient and provider needs. Instead of identifying patients only through the electronic patient registry, care managers also began to utilize direct referrals from providers. In particular, care managers found it more effective to focus their efforts on recruiting patients who are already present in the clinic for other appointments, instead of depending on reaching patients by phone. Responding to provider feedback, care managers now hold sessions with patients immediately before scheduled appointments with their PCP and also offer evening appointments once a week.

We underestimated the demand for additional site level support to assist with overcoming workflow and time management issues after the introduction of the automated devices in the blood pressure intervention. We also discovered the need to collect qualitative and quantitative data to determine if sites were adhering to the established protocol. Responding to these concerns, research staff members were able to offer time-saving techniques, monitor adherence to the protocol, and assist with device maintenance through weekly visits to each of the clinic sites. Additionally, we developed instructional videos to ensure that new clinic staff members are trained in the protocol for proper use of the automated devices.

Many of our initial challenges involved restrictions with data access and slow information technology (IT) development. Our initial approach required clinical data to be transferred from the practices’ EMR to the research team for further analysis to define eligible patients and outcome data. With this approach, we had difficulties obtaining the data we needed in a usable and timely fashion. In addition, delays in the development of the dashboard website pushed back the rollout of the provider education intervention. We eventually moved to an approach that better utilizes the strengths of the community-based practice organization, which has an extensive history of utilizing EMR data to improve clinical quality. The community-based organization became responsible for more of the data abstraction, leveraging their experience with the nuances of their system. Partnering more extensively with members of the organization’s IT department helped to speed the data retrieval and dashboard development processes. The anticipated introduction of a new EMR system across the entire organization in 2013 highlights the importance of continuing to develop these relationships throughout the duration of the project.

Limitations of the study deserve mention. First, it is not mandatory for physicians to view the communications skills training sessions or utilize the elements on the dashboard. This may result in reduced uptake and a potential dilution of the overall effect of the provider education intervention. Additionally, the care management intervention may not be intensive enough to encourage sustained behavior change if patients do not attend all three sessions. Furthermore, in the blood pressure measurement intervention, we are making every attempt to collect high quality data, but are primarily relying on self-reports and qualitative measures to assess adherence to the blood pressure measurement protocol. This will limit the precision with which we can examine adherence. The main limitation for the organizational assessments is that some of our measures will only be collected once, so we will not know how these have changed over the course of the intervention. However, other organizational measures will be collected multiple times, for both intervention and non-intervention sites, allowing estimation of change over time and relative to an unexposed group. Lastly, in a complex healthcare delivery system, additional system-wide or practice-based quality improvement efforts may confound the results, for example, efforts to meet the ‘meaningful use of EMRs’ metrics may improve health system-patient communication. Because data will be evaluated on the system level, other unanticipated factors, in addition to unique clinic level issues, could limit the study’s ability to detect changes in our desired outcomes.

In conclusion, Project ReD CHiP is a multi-faceted, multi-level intervention that targets patients, clinicians, the healthcare organization, and the community to improve hypertension care and reduce racial disparities in blood pressure control. We strengthened the project’s design by tailoring intervention components to meet the needs of the individual clinics. By incorporating principles of community-based participatory research and through the continual engagement of clinic staff, providers, and organizational leaders, planning for sustainability has been a priority of the project. We recognize that its implementation and outcomes are influenced by a variety of factors and have worked to address these barriers. Furthermore, employing a pragmatic trial design and introducing the interventions into uncontrolled, primary care settings enhances the generalizability of our results and could encourage other clinics to incorporate our findings into routine care. Project ReD CHiP will provide knowledge about how integrated multi-level interventions can reduce disparities in blood pressure control; how organizational functioning can affect guideline concordant hypertension care; and how to design sustainable quality improvement interventions in community-based clinical settings.

## Competing interests

The authors declare that they have no competing interests.

## Authors’ contributions

LC, GN, RB, CA, HA, DR, EM, GP, AD, JC, MS, and MH conceived of and designed the study. LC, JM, GN, KC, RB, HA and MH participated in the analysis and interpretation of data. KC provided statistical expertise. LC, JM, SF and KD drafted the article. All authors read and approved the final manuscript.
